# Identification of a Gait Pattern for Detecting Mild Cognitive Impairment in Parkinson’s Disease

**DOI:** 10.3390/s23041985

**Published:** 2023-02-10

**Authors:** Michela Russo, Marianna Amboni, Paolo Barone, Maria Teresa Pellecchia, Maria Romano, Carlo Ricciardi, Francesco Amato

**Affiliations:** 1 Department of Electrical Engineering and Information Technology, University of Naples Federico II, 80125 Naples, Italy; 2 Department of Medicine, Surgery and Dentistry, Scuola Medica Salernitana, University of Salerno, 84081 Baronissi, Italy; 3 IDC Hermitage Capodimonte, 80133 Naples, Italy

**Keywords:** gait analysis, machine learning, Parkinson’s disease, mild cognitive impairment

## Abstract

The aim of this study was to determine a gait pattern, i.e., a subset of spatial and temporal parameters, through a supervised machine learning (ML) approach, which could be used to reliably distinguish Parkinson’s Disease (PD) patients with and without mild cognitive impairment (MCI). Thus, 80 PD patients underwent gait analysis and spatial–temporal parameters were acquired in three different conditions (normal gait, motor dual task and cognitive dual task). Statistical analysis was performed to investigate the data and, then, five ML algorithms and the wrapper method were implemented: Decision Tree (DT), Random Forest (RF), Naïve Bayes (NB), Support Vector Machine (SVM) and K-Nearest Neighbour (KNN). First, the algorithms for classifying PD patients with MCI were trained and validated on an internal dataset (sixty patients) and, then, the performance was tested by using an external dataset (twenty patients). Specificity, sensitivity, precision, accuracy and area under the receiver operating characteristic curve were calculated. SVM and RF showed the best performance and detected MCI with an accuracy of over 80.0%. The key features emerging from this study are stance phase, mean velocity, step length and cycle length; moreover, the major number of features selected by the wrapper belonged to the cognitive dual task, thus, supporting the close relationship between gait dysfunction and MCI in PD.

## 1. Introduction

**Figure 1:**
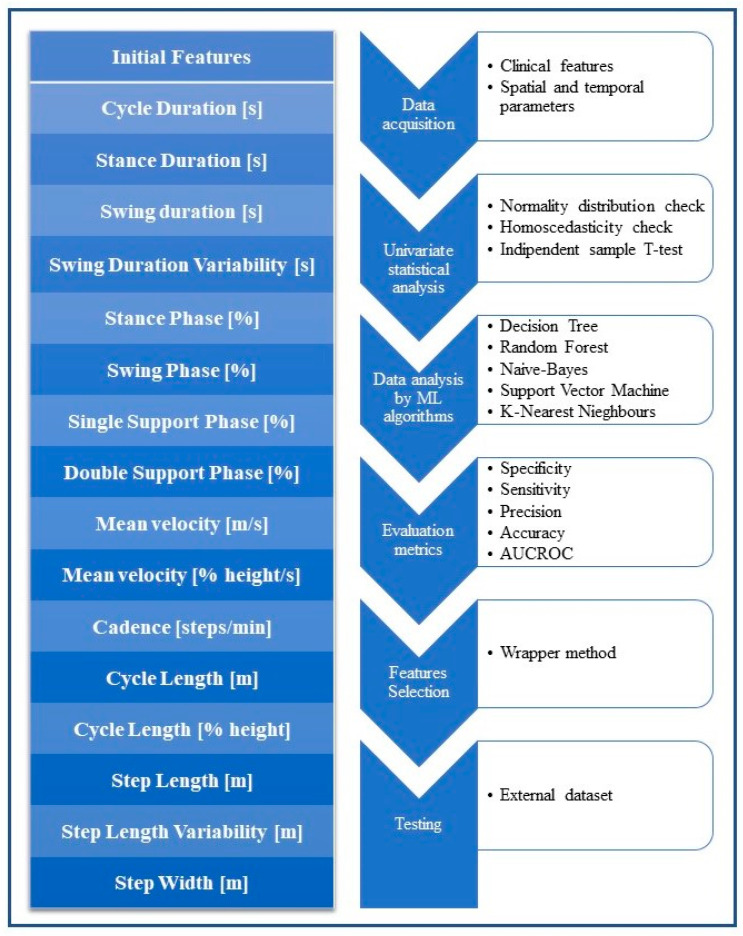
Workflow of the study: the walk of PD patients was acquired in three tasks, the signals were elaborated to extract spatial and temporal features, the features were statistically analyzed and then used as input for implementing ML algorithms.

Parkinson’s disease (PD) is a progressive neurodegenerative condition, with a prevalence of 1–2 out of 1000 people [1]. In PD patients, movements are typically bradykinetic and irregular, gait is characterized by reduced balance and coordination and difficulty in dissociating arms and trunk movements, while, in the advanced stages of the disease, shuffling, festination and freezing episodes are frequently observed [2]. Moreover, non-motor mental symptoms, such as mood and cognitive disorders up to dementia, are commonly observed [3]. In recent years, research has focused on the pre-dementia stages of cognitive impairment in PD, including mild cognitive impairment (PD-MCI). PD-MCI is a syndrome defined by the occurrence of subjective complaints of cognitive decline in combination with the evidence of cognitive dysfunction on neuropsychological examination, without significant interference with functional independence. PD-MCI might involve single or multiple domains (attention, memory, executive functions, language and visuospatial abilities) [4]. While attention, executive and visuospatial dysfunction, possibly accompanied by memory impairment, are common presentations in PD-MCI, increasing language and memory dysfunctions frequently represent the neuropsychological marker of conversion to dementia I [5]. A growing body of evidence suggests an association between cognitive decline and several gait and balance dysfunctions in PD [6,7]. Many studies employing quantitative gait evaluation reported PD-MCI-related gait changes, particularly in terms of reduced stride length and cycle length and increased measures of dynamic instability, such as a longer time in the double support phase and stance phase [8,9]. The identification of gait parameters, which are strongly associated with MCI, could be relevant for early diagnosis. Furthermore, dual-task gait assessment may be more helpful to detect cognition-related gait changes in clinical daily routine [10]. The diagnosis of PD is based on clinical features, generally supported by the use of international evaluation scales, such as the Unified Parkinson’s Disease Rating Scale (UPDRS) and Hoehn and Yahr Scale (H and Y). However, in an effort to improve PD management and move towards a quantitative assessment and recognition of PD motor and non-motor symptoms, which is not possible through the use of the above-mentioned scales, gait analysis may represent a valid aid, since it is a well-established tool for the systematic examination of the way in which a person walks [11]. Gait is typically described by its spatial and temporal parameters, such as step length, step width, stance phase, swing phase and their respective variability [12] (Figure 1). Nowadays, gait sensing technologies, organized in terms of wearable, non-wearable and hybrid approaches, provide a highly consistent framework that explores the issues related to gait analysis [13,14,15,16]. The assessment of gait may be conducted either for clinical purposes or for research. In many institutes of care and scientific research, gait analysis is used for diagnosis, assessment or monitoring the results of treatment.

Machine learning (ML) strategies in the study of human movement have gained popularity as they offer an objective approach to identify or differentiate individuals with movement disorders [17,18].

Using spatial and temporal parameters, PD patients have been classified with 92.6% accuracy using Random Forest and 80.4% using vectorial machine [19]. Using a data mining approach based on gait patterns, it was possible to classify patients affected by PD and atypical Parkinsonism with an accuracy of 86.4% in a dataset of 41 subjects [20]. In addition, ML provides a method to detect the best combination of spatial and temporal parameters of gait able to correctly classify PD patients with or without cognitive impairment [21,22,23,24].

Specifically, our goal in the current study was to determine a subset of spatial and temporal parameters, through an ML approach, in order to define a gait patterm that could reliably distinguish PD patients with MCI from those without it. To this end, we evaluated and trained the ML algorithms to classify PD-MCI on an internal dataset and validated their performance using an external dataset. Figure 1 sets out the workflow of the study.

## 2. Materials and Methods

### 2.1. Participants

The study population consisted of 80 participants consecutively enrolled between January 2018 and March 2022. Participants were selected from patients referred to the Center for Neurodegenerative Diseases of the University of Salerno, Italy. All patients met the Movement Disorder Society (MDS) clinical diagnostic criteria for PD [25] and were recruited according to the following inclusion and exclusion criteria:

Inclusion criteria:

- Hoehn and Yahr score ≤ 3;
- Disease duration ≤ 10 years;
- Age ≥ 45 years;
- Dopaminergic treatment at a stable dosage during the previous 4 weeks;
- Ability to walk independently.

Exclusion criteria:

- Dementia according to the Diagnostic and Statistical Manual of Mental Disorders 5th edition (DSM-V);
- Other neurological diseases;
- Orthopedic diseases;
- Severe cardiovascular/respiratory diseases;
- Anticholinergic or neuroleptic treatment;
- Brain surgery.

A detailed evaluation of demographic, clinical and anthropometric data from each patient was performed. Motor and non-motor symptoms of PD were assessed by means of the Movement Disorder Society Unified Parkinson Disease Rating Scale (MDS-UPDRS). In particular, MDS-UPDRS part I evaluates the non-motor aspects of experiences of daily life, part II assesses the motor aspects of experiences of daily living, whereas part III represents the motor examination and part IV rates the motor complications. An extensive neuropsychological test battery assessing memory, executive/attention, language and visuospatial domains was used to evaluate cognition. While MCI was diagnosed based on the MDS diagnostic criteria for MCI in PD [4], the aim of this study, as mentioned in the previous section, was to recall the association between gait and cognition and to investigate PD-MCI through spatial and temporal features of gait by means of both statistical analysis and an ML approach.

### 2.2. Gait Analysis

**Figure 2:**
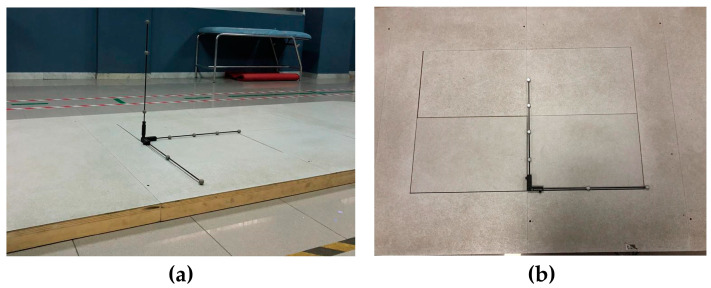
Orthogonal term; (**a**) axes acquisition; (**b**) force platform calibration.

The gait analysis was used to obtain spatial and temporal parameters, which was performed in all patients using a BTS Bioengineering system. The SMART DX is an optical system equipped with six infrared cameras, two video cameras, two force plates, a set of passive markers, an elaborator and data acquisition software (Smart Clinic). First, it requires a phase of calibration of the volume of acquisition and of the force platforms through a specific procedure with an orthogonal term (Figure 2).

Calibration occurs in three phases:

1. Axes acquisition defines the position of the origin and the orientation of the 3 axes of the global reference system (Figure 2a).
2. Wand acquisition defines the volume of acquisition.
3. Force platform calibration identifies the position of force platform (Figure 2b).

**Figure 3:**
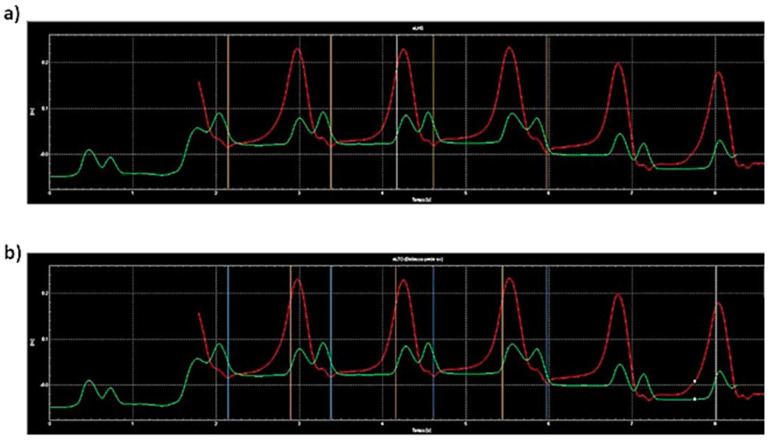
Signal of gait cycle (space on *y*-axis, time on *x*-axis); (**a**) example of the definition of the events of heel strike; (**b**) example of the definition of the events of left toe off. Red and green signals stand for knee and ankle displacements, respectively.

Then, the Davis protocol was applied for all the acquisitions [26], and it consisted of four phases: (a) Anthropometric measurements of the patients (height, weight, leg length, etc.). (b) Positioning of 22 reflective markers on specific points of the body. (c) The standing phase, consisting of assessments of the patient while standing up on a force plate. (d) The walking phase, consisting of the assessments of the patient on a 10 m straight pathway four times. The values of the spatial and temporal parameters were averaged among the four walking phases. The walking phase was evaluated during three different tasks: (1) GAIT: normal gait, namely the single task; (2) MOT: walking while carrying a tray with two glasses filled with water, namely the motor dual task; (3) COG: walking while serially subtracting 7 s starting from 100, namely the cognitive dual task [27]; thus, 12 registrations were acquired for each patient. At the end of each task (GAIT, MOT and COG), the signal of gait cycle was elaborated; the events of gait for the right and left heel strike and toe off were defined, Figure 3. This procedure generated a report for each task from which spatial and temporal parameters were extracted for this study. In order to avoid the influence of the prevalent affected side in PD patients, right and left parameters were averaged.

### 2.3. Tool and Algorithms

Univariate statistical analysis was employed to assess the clinical features and spatial and temporal parameters of PD patients with and without MCI. IBM SPSS (v.27) was used to perform all the statistical analyses. Data normality and variance homogeneity were verified using the Kolmogorov–Smirnov test and Levene’s test, respectively. A *t*-test for independent samples was employed when both the previous assumptions were verified; a Mann–Whitney U test was otherwise employed. The level of significance was set at a *p*-value < 0.05 for all statistical analyses.

**Figure 4:**
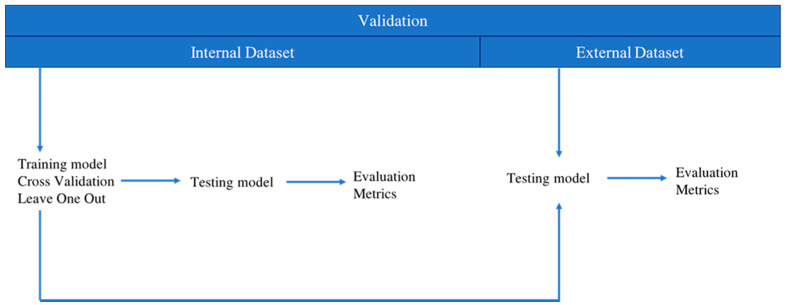
Two types of validation methods: internal and external dataset.

Afterward, a supervised learning analysis was performed through Knime analytics platform (v.4.4.2), which is a well-known platform in the literature for performing ML analyses by creating a workflow with a combination of nodes; it has already been employed in several biomedical studies [28,29,30,31]. Two analyses were carried out using spatial and temporal parameters of gait cycle: (1) supervised algorithms were implemented for spatial and temporal parameters of each task; (2) a feature selection method was implemented on the spatial and temporal parameters using, as input, all three tasks to reduce the dimensionality of the dataset; both analyses were performed on one internal validation dataset, composed of 60 patients, and were tested on an external validation dataset, composed of 20 patients who were not considered in the implementation of the previous models, with the aim of evaluating the performance of such models (Figure 4).

Five ML algorithms were chosen to detect the presence of MCI in the analyzed patients [32]:

Decision Tree (DT) is widely used for categorized problems and its flow structure makes it simple to understand. DT is a classification algorithm that groups the instances in a recursive manner. The roots of the tree represent the nodes where the instances are split. The leaves are the intermediate and final classes where the instances are assigned. Based on the results of the tests along the path, instances are classified by traveling them from the root of the tree down to a leaf [32]. DT has more advantages over the other supervised methods, which require less effort for data preparation during the pre-processing phase; indeed, the normalization of data is not always required. DT is typically preferred in many fields, such as healthcare.

Random Forest (RF) is a combination of a large number of decision trees and it is a well-known classifier due to its stability and its ability to maintain accuracy when a considerable proportion of data is missing. RF is based on the bagging algorithm and uses the ensemble learning technique. It creates many trees on the subset of the data and combines the output of all such trees. In this way, it reduces the overfitting problem of the DT algorithm and reduces the variance, therefore, improving the accuracy. RF is usually robust to outliers and can handle them automatically; however, it requires much more time and computational power to train the dataset [33].

Naïve Bayes (NB) is a model based on Bayes’ theorem of probability to predict the class of unknown datasets with a fundamental assumption: each feature makes an independent and equal contribution to the outcome.

NB is a fast and easy ML algorithm used to predict a class of datasets and can be used for binary as well as multi-class classification. It relies on the assumption, rarely satisfied in real life, that all predictors are independent; this fact limits the applicability of such an algorithm in some real-world cases [34].

Support Vector Machine (SVM) is an ML method that has become exceedingly popular for simplicity and flexibility for addressing a range of classification problems. The objective of the SVM is to find a hyperplane in *N*-dimensional space that distinctly classifies the data points. To separate the two classes of data points, there are many possible hyperplanes, and the dimension of the hyperplane depends on the number of features. If the number of input features is 2, the hyperplane is just a line; if the number of input features is 3, the hyperplane becomes a two-dimensional plane [35].

K-Nearest Neighbor (KNN) algorithm assumes that similar data are near to each other. KNN is calculated on the basic value of k, which defines how many nearest neighbors should be considered to define the class of a training sample data point. There is no particular way to determine the best value for k; therefore, some values were experimented with to find the best of them. A very low value for k, such as k = 1 or k = 2, can be noisy and lead to the presence of outliers in the model. On the other hand, large values for k are good in principle, but the algorithm may find some computational difficulties. For these reasons, in general, it may be hard to determine the optimal value of k [36].

Moreover, a leave-one-out cross-validation was applied to estimate the skill of an ML model and to reduce overfitting: this procedure split the dataset into a training set and a testing set, using only one observation as part of the testing set. The data in the training set were used to build the model, which predicts the response value of the one observation left out of the training set. This process was repeated *n* times, where *n* is the total number of observations, leaving out a different observation from the training set each time [37].

Various evaluation metrics were used to assess the performance of the proposed supervised algorithms [38]:

- Specificity: capacity to correctly detect subjects not belonging to the group under examination:

(1) $$\mathrm{Specificity}=\frac{\mathrm{TN}}{\mathrm{TN}+\mathrm{FP}\;}$$

- Sensitivity: capacity to correctly detect subjects belonging to the group under examination:

(2) $$\mathrm{Sensitivity}\;=\frac{\mathrm{TP}}{\mathrm{TP}+\mathrm{FN}\;}$$

- Precision: a measure of the positive patterns correctly predicted from the total predicted patterns in a positive class:

(3) $$\;\mathrm{Precision}=\frac{\mathrm{TP}}{\mathrm{TP}+\mathrm{FP}\;}$$

- Accuracy: the ratio of correct predictions over the total number of records:
  (4) $$\mathrm{Accuracy}=\frac{\mathrm{TP}+\mathrm{TN}}{\mathrm{TN}+\mathrm{FP}+\mathrm{FN}+\mathrm{TP}}$$
  where TN is the number of true negatives, TP represents the number of true positives predicted by the classifier, FP is the number of false positives, and, consequently, FN is the number of false negatives.

- Area Under the Curve Receiver Operating Characteristic (AUCROC): a qualitative indicator for the binary classification, ranging from 0 to 1, with 0.5 indicating a classification not better than random guessing.

Subsequently, a wrapper method was performed to identify the best subset of characteristics, which improved the classification accuracy for each ML algorithm. The wrapper feature selection method initiates training by using a subset of characteristics and then appends or deletes a characteristic using a selection criterion [39]. The selection criterion directly measures the change in model performance that results from appending or deleting a characteristic. The algorithm replicates training, improving a model until its stopping criteria are satisfied. Finally, the ML algorithms’ performances were evaluated on an external validation dataset.

## 3. Results

Univariate statistical analysis was conducted on clinical–demographic features and on spatial and temporal parameters of each single task. The statistical analysis was employed both on the internal dataset and on the external dataset. The internal dataset was composed of 31 patients classified as PD-MCI and 29 as PD cognitively unimpaired (PD-NO MCI). As expected, significant differences emerged, both in clinical–demographic features and in spatial temporal parameters. In particular, as shown in Table 1 and expected [40], PD-MCI patients were older, with a more advanced stage and more severe motor symptoms, as indicated by higher Hoehn and Yahr Scale and MDS-UPDRS part III, respectively.

Concerning the external dataset, composed of 9 patients with and 11 patients without MCI, univariate analysis was conducted on clinical–demographic features. In particular, as shown in Table 2, the variables were properly matched, and no significant statistical differences were found. Moreover, in order to show the coherence between the internal and external datasets, a comparison of the classes (PD-MCI and PD-NO MCI) between the datasets was performed. To this end, univariate analysis on clinical and demographic variables was carried out on the PD patients with and without MCI, belonging to both datasets, showing that no statistically significant difference occurred (see the Supplementary Materials; Tables S1 and S2).

Moreover, the two groups differed in all three conditions for different spatial temporal parameters, as summarized in Table 3. In the GAIT single task, PD-MCI showed worse gait parameters than PD-NO MCI. In detail, PD-MCI exhibited reduced step length (*p* < 0.009), cycle length (*p* < 0.007), velocity (*p* < 0.037) and longer stance phase (*p* < 0.020), mainly due to the increased double support phase (*p* < 0.034). In the MOT dual task, PD-MCI continued to show reduced step length (*p* < 0.008), cycle length (*p* < 0.017) and velocity (*p* < 0.045). In addition, PD-MCI showed both reduced swing phase (*p* < 0.010) and single support phase (*p* < 0.007). In the COG dual task, PD-MCI again showed the same gait pattern displayed during the previous two tasks but, noteworthily, with an increased level of statistical significance.

Concerning the ML analysis, all the evaluation metrics of the best two classifiers for each task are summarized in Table 4.

RF classifier showed the best performance in each task on the external validation dataset; RF reached the highest values in terms of accuracy (65.0% in task GAIT, 70.0% in task MOT and 71.4% in task COG), sensitivity (90.0% in task GAIT, 80.0% in task MOT and 70.0% in task COG) and precision (60.0% in task GAIT, 66.7% in task MOT and 70.0 in task COG). However, it is possible that the AUCROC of RF was the lowest if compared with the other classifiers. Indeed, SVM yielded the highest cumulative accuracy in both validation datasets (0.784 and 0.773, respectively), followed by the KNN with 0.720 on the external validation dataset.

In order to identify the main spatial and temporal parameters for the classification of PD-MCI, the wrapper feature selection method allowed us to reduce the number of features. As a result, the prior 51 spatial and temporal parameters were reduced to 17. The feature selection subset for each classification algorithm and the evaluation metrics are shown in Table 5. Among the 17 features selected, the major number belonged to the COG dual task. In particular, the cycle length, the step length and its variability were selected by several different algorithms: DT, RF, NB and KNN. The other features selected by the wrapper method were divided between the other two tasks: swing duration and its variability, cadence and step length and width for the single task and swing duration variability and cadence for the motor dual task. Among the tested algorithms, NB picked the highest number of features (six), with the highest accuracy on the external dataset (85.7%), followed by RF, which selected four features and reached an accuracy of 81.0%. Conversely, despite picking the lowest number of features (two), the SVM and the KNN achieved an accuracy of 81.0%. 

## 4. Discussion

The main aim of the present study was to verify, and then to validate in an independent cohort, if ML algorithms, implemented with gait analysis variables, can distinguish PD patients with MCI from those without it. To this end, firstly, spatial and temporal parameters were acquired in a motion analysis laboratory in three different gait conditions for each patient. Subsequently, univariate statistical analysis and ML analysis were implemented on all gait parameters. Furthermore, the wrapper approach for feature selection and optimized different ML classifiers were employed in order to catch the PD-MCI from the PD-NO MCI. Furthermore, all the ML analyses were tested on an internal dataset of 60 patients and validated on an external dataset of 20 patients. In the present study, coherently with a recent meta-analysis [40], PD-MCI patients were older and affected by more severe motor symptoms, reflected by a higher H and Y stage and an increased motor score for the MDS-UPDRS III (MDS-UPDRS III). As reported in the Methods section, we consecutively enrolled PD patients, fulfilling inclusion and exclusion criteria, without “a priori” selection, thus, including a PD population representing, as much as possible, the natural course of the disease. This procedure obviously brings out the clinical variables commonly associated with PD-MCI, without affecting, in our opinion, the clinical significance of the present results. Furthermore, consistently with previous findings [6,41,42], we confirm that PD-MCI, as compared with PD-NO MCI, showed worse gait performance and reduced dynamic balance, especially under the dual task condition, with a subsequent increased risk of falling. In particular, compared with PD-NO MCI, PD-MCI patients displayed reduced step length, cycle length and velocity and increased double support phase and stance phase. Although, PD-MCI versus PD-NO MCI patients showed significant differences in all three tasks, displaying more dysfunctions during the COG task. These findings further support the tight connection between walking performance and cognitive dysfunction in PD, where the magnitude of the dual task interference on gait in PD appears to be directly related to the severity of the underlying cognitive dysfunction [7]. Additionally, in line with previous research [22,43,44], our ML algorithms detected PD-MCI with an accuracy of over 80.0%. Despite obtaining comparable accuracy, our algorithm recognized PD-MCI with lower sensitivity.

In a previous study, spatial and temporal parameters were used to classify MCI in PD patients by implementing three ML algorithms, but the dataset was composed of 45 subjects, no investigative statistical analysis was performed, the Synthetic Oversampling technique was employed to augment the dataset through artificial data and there was no validation in the ML analysis [21].

Similarly, but using a different protocol, Chen et al. distinguished PD-MCI patients from subjects with MCI without PD, by gait and jump parameters using three conditions with BTS G-WALK. The obtained gait parameters were trained with the SVM classification model, reaching an accuracy of 91.67% [22]. Indeed, the very good accuracy of these models might denote gait differences between subjects with and without PD rather than gait patterns specifically related to the type of MCI. In addition, very recently, Ghoraani and collaborators determined the gait features that are important in developing an ML-based detection of healthy, MCI (of unspecified nature) and Alzheimer’s disease groups [43]. Similarly, Boettcher et al. developed an ML approach to analyze gait data during a dual task condition in order to distinguish subjects with any type of cognitive impairment from healthy individuals [44].

In contrast with such research that included subjects with cognitive impairment of disparate origin, the present study focused on a more homogeneous cohort, namely PD patients, categorized in two subgroups based on the presence of MCI, thus, allowing us to attribute the difference between the two groups to the presence of MCI. In addition, it is worth noting that, differently from the above-mentioned findings, we achieved comparable evaluation metrics but obtained a subset of specific gait features, even on an external validation dataset, thus, evidently increasing the reliability of our results. Indeed, another strength of this study is the presence of a good number of subjects, i.e., 80, whose gait was acquired through an optoelectronic system.

Indeed, to our knowledge, this study is the first one aiming to identify a subset of quantitative gait features, through an ML method, with the scope of detecting PD patients with MCI, using validation on an external dataset. It is worth noting that the results obtained on the external dataset were better than the results on the internal dataset; this supports the generalization ability of the learned model on the internal dataset and the absence of over-fitting in the training phase.

The present study, of course, has some limitations. The analysis focused only on gait patterns, i.e., spatial and temporal parameters extracted by gait, but an extension of the work could consist of also extracting kinetic and kinematic features to perform the same type of classification in order to investigate the differences that might come out. Similarly, patients could perform other postural exercises (i.e., sway) to test the feasibility of other features. Finally, as the performance of ML algorithms was widely influenced by feature selection, the efficacy of different feature extraction algorithms, such as maximum relevance and minimum redundancy strategy, could be evaluated on gait parameters.

## 5. Conclusions

The present study further supports the close relationship between gait dysfunction and MCI in PD. In addition, importantly, it shows that selected gait features might serve to implement ML algorithms able to detect PD-MCI, even on an independent dataset. Recently, Landolfi et al. reported, in their systematic review, that the discrimination between PD subtypes has not been studied enough and there has been scarce comparability of the outcomes due to the heterogeneous patients’ selections and small sample size, usually made up for using the cross-validation technique, a process that could create data overfitting [18]. In this study, we overcame all of these main limitations from previous research by enlarging the sample size, selecting homogeneous patients and introducing the test on a completely external set of patients. In conclusion, this research still supports that peculiar quantitative gait features, such as stance phase, mean velocity, step length and cycle length, may represent a surrogate biomarker for cognitive impairment in PD.

## Figures and Tables

**Table 1:** Clinical–demographic features of PD patients with (PD-MCI) and without (PD-NO MCI) mild cognitive impairment belonging to internal dataset (mean ± standard deviation).

	PD-MCI (Sample Size = 31)		PD-NO MCI (Sample Size = 29)		
Variables	Mean	SD	Mean	SD	*p*-Value
Age	66.20	8.50	61.32	8.10	0.015 *
BMI	28.30	3.98	26.98	3.09	0.429
Disease Duration	5.41	2.44	4.74	2.64	0.545
LEDD	576.60	378.91	554.81	442.98	0.901
Hoehn &Yahr	1.95	0.30	1.76	0.41	0.045 *
MDS-UPDRS: Part I	9.23	6.89	7.20	4.36	0.070
MDS-UPDRS: Part II	8.10	5.50	7.98	6.31	0.524
MDS-UPDRS: Part III	25.56	8.61	20.98	7.67	0.041 *
MDS-UPDRS: Part IV	1.50	2.93	2.10	3.15	0.434

* Significance level at 0.05. Significant *p*-values are highlighted in bold.

**Table 2:** Clinical–demographic features of PD patients with (PD-MCI) and without (PD-NO MCI) mild cognitive impairment belonging to external dataset (mean ± standard deviation).

	PD-MCI (Sample Size = 9)		PD-NO MCI (Sample Size = 11)		
Variables	Mean	SD	Mean	SD	*p*-Value
Age	71.33	7.57	63.55	11.19	0.092
BMI	28.91	4.49	24.48	3.31	0.134
Disease Duration	3.83	1.76	4.18	3.10	0.768
LEDD	718.13	567.98	569.55	569.24	0.581
Hoehn &Yahr	2.06	0.17	1.90	0.17	0.210
MDS-UPDRS: Part I	9.56	5.70	9.73	5.04	0.944
MDS-UPDRS: Part II	10.11	2.93	10.91	8.79	0.798
MDS-UPDRS: Part III	23.78	4.74	22.91	9.77	0.810
MDS-UPDRS: Part IV	0.67	1.32	2.09	3.17	0.226

**Table 3:** Univariate statical analysis of spatial and temporal parameters of each task.

GAIT TASK	PD-MCI (Sample Size = 40)		PD-NO MCI (Sample Size = 40)		
Parameters	Mean	SD	Mean	SD	*p*-Value
Cycle Duration [s]	1.12	0.13	1.10	0.11	0.532
Stance Duration [s]	0.68	0.09	0.66	0.07	0.279
Swing duration [s]	0.43	0.04	0.44	0.04	0.738
Swing Duration Variability [s]	0.03	0.02	0.04	0.06	0.382
Stance Phase [%]	61.14	1.87	60.04	2.29	0.020 *
Swing Phase [%]	38.88	1.87	39.52	1.85	0.128
Single Support Phase [%]	38.89	1.87	39.36	2.61	0.353
Double Support Phase [%]	11.47	3.15	10.25	1.71	0.034 *
Mean velocity [m/s]	0.97	0.18	1.05	0.16	0.037 *
Mean velocity [%height/s]	58.28	10.95	62.48	9.56	0.072
Cadence [steps/min]	108.91	11.62	108.83	12.15	0.976
Cycle Length [m]	1.06	0.15	1.15	0.15	0.007 **
Cycle Length [%height]	64.01	9.97	68.66	7.79	0.023 *
Step Length [m]	0.48	0.12	0.55	0.10	0.009 **
Step Length Variability [m]	0.25	0.49	0.15	0.37	0.289
Step Width [m]	0.09	0.05	0.09	0.04	0.536
MOT TASK	PD-MCI (N = 40)		PD-NO MCI (N = 40)		
Cycle Duration [s]	1.09	0.13	1.08	0.12	0.754
Stance Duration [s]	0.67	0.10	0.65	0.08	0.340
Swing duration [s]	0.45	0.17	0.43	0.05	0.567
Swing Duration Variability [s]	0.03	0.02	0.03	0.02	0.568
Stance Phase [%]	61.45	2.34	60.37	1.74	0.018 *
Swing Phase [%]	38.54	2.34	39.74	1.63	0.010 *
Single Support Phase [%]	38.53	2.31	39.80	1.75	0.007 **
Double Support Phase [%]	12.11	2.75	11.38	3.21	0.276
Mean velocity [m/s]	0.95	0.20	1.04	0.17	0.045 *
Mean velocity [%height/s]	57.30	11.58	61.77	9.63	0.064
Cadence [steps/min]	110.98	11.49	111.95	11.76	0.710
Cycle Length [m]	1.02	0.17	1.11	0.17	0.017 *
Cycle Length [%height]	61.75	10.59	66.39	9.10	0.039 *
Step Length [m]	0.48	0.11	0.54	0.09	0.008 **
Step Length Variability [m]	0.19	0.50	0.08	0.15	0.193
Step Width [m]	0.09	0.05	0.09	0.04	0.876
COG TASK	PD-MCI (N = 40)		PD-NO MCI (N = 40)		
Cycle Duration [s]	1.20	0.20	1.15	0.14	0.122
Stance Duration [s]	0.76	0.15	0.70	0.09	0.040 *
Swing duration [s]	0.45	0.06	0.44	0.05	0.647
Swing Duration Variability [s]	0.05	0.05	0.03	0.02	0.071
Stance Phase [%]	63.07	2.51	61.36	2.17	0.002 **
Swing Phase [%]	37.91	5.07	38.58	2.18	0.447
Single Support Phase [%]	37.25	2.58	38.27	2.92	0.101
Double Support Phase [%]	14.09	3.90	11.73	1.96	0.001 **
Mean velocity [m/s]	0.79	0.19	0.95	0.17	0.000 ***
Mean velocity [%height/s]	48.29	11.71	56.70	10.53	0.001 **
Cadence [steps/min]	102.16	14.50	106.33	12.77	0.175
Cycle Length [m]	0.93	0.18	1.07	0.15	0.000 ***
Cycle Length [%height]	56.74	12.37	63.74	8.43	0.004 **
Step Length [m]	0.42	0.11	0.52	0.10	0.000 ***
Step Length Variability [m]	0.28	0.45	0.08	0.22	0.015 *
Step Width [m]	0.10	0.06	0.11	0.12	0.636

Significant *p*-values are highlighted in bold. * Significance level at 0.05. ** Significance level at 0.01. *** Significance level at 0.001.

**Table 4:** Evaluation metrics on internal and external validation dataset for each task.

		Task GAIT		Task MOT		Task COG	
Evaluation Metrics	Dataset	RF	KNN	RF	DT	RF	SVM
AUCROC	Internal	0.557	0.616	0.623	0.658	0.500	0.784
	External	0.555	0.720	0.655	0.680	0.518	0.773
Accuracy	Internal	55.0	61.7	63.3	63.3	70.0	73.3
	External	65.0	57.1	70.0	61.9	71.4	66.7
Sensitivity	Internal	54.8	67.7	61.3	51.6	74.2	71.0
	External	90.0	70.0	80.0	50.0	70.0	60.0
Specificity	Internal	58.6	58.6	65.5	75.9	65.5	75.9
	External	40.0	45.5	60.0	72.7	72.7	72.7
Precision	Internal	57.1	62.5	65.5	69.6	69.7	75.9
	External	60.0	53.8	66.7	62.5	70.0	66.7

**Table 5:** Feature selection by the wrapper method. “g” is for GAIT, “m” is for MOT and “c” is for COG; 95% confidence intervals are enclosed in brackets.

Classifier	Features Selection	Internal Dataset Accuracy	External Dataset		
			Accuracy	Sensitivity	Specificity
**DT**	g_Swing Duration g_Swing Duration Variability c_Step Length Variability	67.7 (43.7–3.7)	79.1 (57.4–90.1)	70.0 (39.7–89.2)	90.0 (59.6–98.2)
**RF**	g_Step Length g_ Step Width c_ Step Length c_ Step Width	72.3 (49.1–85.7)	81.0 (60.0–92.3)	60.0 (31.3–83.2)	100.0 (72.3–100)
**NB**	g_Cadence m_Swing Duration Variability c_Stance Phase c_ Cycle Length c_Step Length c_Step Length Variability	2.3 (49.1–85.7)	85.7 (65.4–95.0)	70.1 (39.7–89.2)	100.0 (72.3–100)
**SVM**	m_Mean Velocity c_ Mean Velocity	77.8 (54.8–91.0)	81.0 (60.0–92.3)	70.0 (39.7–89.2)	90.0 (59.6–98.2)
**KNN**	m_Swing Duration Variability c_ Cycle Length	61.2 (38.6–79.7)	81.0 (60.0–92.3)	80.0 (49.0–94.3)	80.0 (49.0–94.3)

## Data Availability

The data presented in this study are available on request from the corresponding author. The data are not publicly available due to the privacy policy.

## Supplementary Materials

The following supporting information can be downloaded at: https://www.mdpi.com/article/10.3390/s23041985/s1, Table S1: Clinical-demographic features of PD patients with mild cognitive impairment (PD-MCI) belong to internal dataset and external dataset (mean ± standard deviation); Table S2: Clinical-demographic features of PD patients without mild cognitive impairment (PD-noMCI) belong to internal dataset and external dataset (mean ± standard deviation); Table S3: Confidence interval for accuracy, sensitivity, specificity and precision of each algorithm.
