# Supplementary material for: Identification of a Gait Pattern for Detecting Mild Cognitive Impairment in Parkinson’s Disease
Source: Sensors (Basel). 2023 Feb 10;23(4):1985. doi: 10.3390/s23041985 (PMC9963713; doi:10.3390/s23041985)
Supplement: Supplementary file 1 [file sensors-23-01985-s001.zip › sensors-2106058-supplementary.pdf]

sensors

Article

Identification of a Gait Pattern for Detecting Mild Cognitive Impairment in Parkinson's Disease

Michela Russo [1], Marianna Amboni [2,3], Paolo Barone [2], Maria Teresa Pellecchia [2], Maria Romano [1], Carlo Ricciardi [1,*,†] and Francesco Amato [1,†]

[1] Department of Electrical Engineering and Information Technology, University of Naples Federico II, 80125 Naples, Italy
[2] Department of Medicine, Surgery and Dentistry, Scuola Medica Salernitana, University of Salerno, 84081 Baronissi, Italy
[3] IDC Hermitage Capodimonte, 80133 Naples, Italy
* Correspondence: carlo.ricciardi@unina.it
† These authors contributed equally to this work.

Table S1. Clinical-demographic features of PD patients with mild cognitive impairment (PD-MCI) belong to internal dataset and external dataset (mean ± standard deviation).

	**PD-MCI/Internal dataset (sample size=31)**		**PD-MCI/External dataset (sample size=9)**		
**Variables**	**Mean**	**SD**	**Mean**	**SD**	***p*-value**
Age	66.20	8.50	71.33	7.57	0.106
BMI	28.30	3.98	28.91	4.49	0.524
Disease Duration	5.41	2.44	3.83	1.77	0.167
LEDD	576.60	378.91	718.13	576.98	0.825
Hoehn &Yahr	1.95	0.30	2.06	0.17	0.406
MDS-UPDRS-TOT	43.03	8.85	44.11	7.22	0.276

*Significance level at 0.05.

Table S2. Clinical-demographic features of PD patients without mild cognitive impairment (PD-noMCI) belong to internal dataset and external dataset (mean ± standard deviation).

	**PD-noMCI/Internal dataset (sample size=29)**		**PD-noMCI/External dataset (sample size=11)**		
**Variables**	**Mean**	**SD**	**Mean**	**SD**	***p*-value**
Age	61.32	8.10	63.55	11.19	0.417
BMI	26.98	3.09	24.48	3.31	0.272
Disease Duration	4.74	2.64	4.18	3.10	0.369
LEDD	554.81	442.98	569.55	569.24	0.550
Hoehn &Yahr	1.76	0.41	1.90	0.17	0.196
MDS-UPDRS - TOT	35.45	11.65	41.55	18.15	0.308

*Significance level at 0.05.

Citation: Russo, M.; Amboni, M.; Barone, P.; Pellecchia, M.T.; Romano, M.; Ricciardi, C.; Amato, F. Identification of a Gait Pattern for Detecting Mild Cognitive Impairment in Parkinson's Disease. *Sensors* **2023**, *23*, 1985. https://doi.org/10.3390/s23041985

Academic Editor: Giovanni Saggio

Received: 9 December 2022
Revised: 4 January 2023
Accepted: 7 February 2023
Published: 10 February 2023

Copyright: © 2023 by the authors. Licensee MDPI, Basel, Switzerland. This article is an open access article distributed under the terms and conditions of the Creative Commons Attribution (CC BY) license (https://creativecommons.org/licenses/by/4.0/).

Table S3. Confidence interval for accuracy, sensitivity, specificity and precision of each algorithm.

Task GAIT					
**Evaluation metrics**	**Dataset**	**RF**	**CI**	**KNN**	**CI**
**Accuracy**	**Internal**	55.0	43.0% to 67.7%	61.7	49.0% to 72.9%
	**External**	65.0		57.1	
**Sensitivity**	**Internal**	54.8	35.1% to 68.0%	67.7	46.9% to 78.8%
	**External**	90.0		70.0	
**Specificity**	**Internal**	58.6	41.7% to 75.4%	58.6	40.7% to 74.5%
	**External**	40.0		45.5	
**Precision**	**Internal**	57.1	39.0% to 74.5%	62.5	45.3% to 77.1%
	**External**	60.0		53.8	

Task MOT					
**Evaluation metrics**	**Dataset**	**RF**	**CI**	**DT**	**CI**
**Accuracy**	**Internal**	63.3	47.3% to 71.4%	63.3	51.6% to 0.74.4%
	**External**	70.0		61.9	
**Sensitivity**	**Internal**	61.3	40.7% to 73.6%	51.6	34.8% to 68.0%
	**External**	80.0		50.0	
**Specificity**	**Internal**	65.5	44.0% to 77.3%	75.9	57.9% to 87.8%
	**External**	60.0		72.7	
**Precision**	**Internal**	65.5	44.0% to 77.3%	69.6	49.1% to 84.4%
	**External**	66.7		62.5	

Task COG					
**Evaluation metrics**	**Dataset**	**RF**	**CI**	**SVM**	**CI**
**Accuracy**	**Internal**	70.0	55.8% to 78.7%	73.3	55.8% to 78.7%
	**External**	71.4		66.7	
**Sensitivity**	**Internal**	74.2	53.4% to 83.9%	71.0	53.4% to 83.9%
	**External**	70.0		60.0	
**Specificity**	**Internal**	65.5	47.3% to 80.1%	75.9	57.8% to 87.8%
	**External**	72.7		72.7	
**Precision**	**Internal**	69.7	51.4% to 82.0%	75.9	57.8 % to 87.8%
	**External**	70.0		66.7	

Disclaimer/Publisher's Note: The statements, opinions and data contained in all publications are solely those of the individual author(s) and contributor(s) and not of MDPI and/or the editor(s). MDPI and/or the editor(s) disclaim responsibility for any injury to people or property resulting from any ideas, methods, instructions or products referred to in the content.
